# Supplementary material for: Empirical methods for controlling false positives and estimating confidence in ChIP-Seq peaks
Source: BMC Bioinformatics. 2008 Dec 5;9:523. doi: 10.1186/1471-2105-9-523 (PMC2628906; doi:10.1186/1471-2105-9-523)
Supplement: Additional File 1 — A variety of html documents from the USeq web site detailing the available applications, their best usage, output file type descriptions, command line menus, etc. [file 1471-2105-9-523-S1.zip › USeqUserGuides/JavaDoc.zip!/JavaDoc/util/gen/HTML.html]

HTML


|  |  |  |
| --- | --- | --- |
| **Overview** \| **Package** \| **Class** \| **Use** \| **Tree** \| **Deprecated** \| **Index** \| **Help** | |  |
| **PREV CLASS**   **NEXT CLASS** | **FRAMES**    **NO FRAMES**     **All Classes** |  |
| SUMMARY: NESTED \| FIELD \| CONSTR \| METHOD | DETAIL: FIELD \| CONSTR \| METHOD |  |


---


## util.gen Class HTML

```
java.lang.Object
  util.gen.HTML
```

---

``` public class HTML extends java.lang.Object ```

Static methods for HTML related tasks.

---

**Constructor Summary**

`HTML()`


| **Method Summary** | |
| --- | --- |
| `static java.lang.String` | `fetchArrowButton(java.lang.String formParamToSet, java.lang.String value, java.lang.String pathToArrowGifs, int buttonIncrement)`             Returns an upDown arrow ala Mac look and feel that will set a formParm to a given value and submit the form provided the form is named "form" (ie  `static java.lang.String` \| `fetchCheckGroup(java.lang.String name, java.lang.String toCheck, java.lang.String[] boxes)`             Returns a selected check box group with the toSelect selected if present in the String[] \| |
| `static java.lang.String` | `fetchMultipleOptionGroup(java.lang.String[] toSelect, java.lang.String[] options)`             Returns a selected option group with the toSelect selected if present in the String[] of options. |
| `static java.lang.String` | `fetchOptionGroup(java.lang.String toSelect, java.lang.String[] options)`             Returns a selected option group with the toSelect selected if exactly matches one of the String[] options |
| `static java.lang.String` | `fetchOptionGroupLoose(java.lang.String toSelect, java.lang.String[] options)`             Returns a selected option group with the toSelect selected if at all present in the String[] options. |
| `static java.lang.String` | `fetchRadio(java.lang.String name, java.lang.String value, java.lang.String potential)`             Returns a radio input line with CHECKED if the value is equal to the potential |
| `static java.lang.String` | `fetchRadioGroup(java.lang.String name, java.lang.String toCheck, java.lang.String[] boxes)`             Returns a selected radio group with the toSelect selected if present in the String[] |
| `static java.lang.String` | `fetchRadioGroupSubmit(java.lang.String name, java.lang.String toCheck, java.lang.String[] boxes, java.lang.String nbsp)`             Returns a selected radio group with the toSelect selected if present in the String[] Also throws in a little javascript to submit the form if the button is clicked. |
| `static java.lang.String` | `fetchValueOptionGroup(java.lang.String toSelect, java.lang.String[] options)`             Returns a selected option group with the toSelect selected if present in the String[]. |
| `static java.lang.String` | `filterField(java.lang.String field, int lengthCutOff)`             Filters a String for bad characters replacing them with html equivalents. |
| `static java.lang.String[]` | `filterFields(java.lang.String[] fields, int lengthCutOff)`             Filters a String[] for bad characters replacing them with html equivalents. |
| `static java.lang.String` | `makeArrowedTableRow(java.lang.String[] headings, java.lang.String[] values)`             Example table row with arrow buttons. |
| `static java.util.HashMap` | `parseMultiPartRequest(javax.servlet.http.HttpServletRequest request)`             Parses a multipart request returning a HashMap of fieldName: String, if the same fieldName is found more than once, a String[] is created. |
| `static java.lang.String[]` | `parseStringArray(java.lang.Object obj)`             For extracting either a String[] or making a new String[] by casting the object as a String. |
| `static void` | `populateBean(java.lang.Object bean, javax.servlet.http.HttpServletRequest request)`             Fills in all the params of a bean. |

**Methods inherited from class java.lang.Object**

`equals, getClass, hashCode, notify, notifyAll, toString, wait, wait, wait`

**Constructor Detail**

### HTML

```
public HTML()
```


**Method Detail**

### parseMultiPartRequest

```
public static java.util.HashMap parseMultiPartRequest(javax.servlet.http.HttpServletRequest request)
```

:   Parses a multipart request returning a HashMap of fieldName: String,
    if the same fieldName is found more than once, a String[] is created.
    Files are referenced by their fieldName : FileItem obj. Call FileItem.write(File) to save
    it to disk. Assumes file field names are always unique.

---


### parseStringArray

```
public static java.lang.String[] parseStringArray(java.lang.Object obj)
```

:   For extracting either a String[] or making a new String[] by casting the object as a String.

---


### filterField

```
public static java.lang.String filterField(java.lang.String field,
                                           int lengthCutOff)
```

:   Filters a String for bad characters replacing them with html equivalents.
    Truncates the string to a lengthCutOff

---


### filterFields

```
public static java.lang.String[] filterFields(java.lang.String[] fields,
                                              int lengthCutOff)
```

:   Filters a String[] for bad characters replacing them with html equivalents.
    Truncates the Strings to a lengthCutOff. Modifies original String[]

---


### fetchArrowButton

```
public static java.lang.String fetchArrowButton(java.lang.String formParamToSet,
                                                java.lang.String value,
                                                java.lang.String pathToArrowGifs,
                                                int buttonIncrement)
```

:   Returns an upDown arrow ala Mac look and feel that will set a formParm to a given value and
    submit the form provided the form is named "form" (ie 

    ### makeArrowedTableRow

    ```
    public static java.lang.String makeArrowedTableRow(java.lang.String[] headings,
                                                       java.lang.String[] values)
    ```

    :   Example table row with arrow buttons.

    ---


    ### fetchOptionGroup

    ```
    public static java.lang.String fetchOptionGroup(java.lang.String toSelect,
                                                    java.lang.String[] options)
    ```

    :   Returns a selected option group with the toSelect selected if exactly matches one of the String[] options

    ---


    ### fetchOptionGroupLoose

    ```
    public static java.lang.String fetchOptionGroupLoose(java.lang.String toSelect,
                                                         java.lang.String[] options)
    ```

    :   Returns a selected option group with the toSelect selected if at all present in the String[] options.
        Case-insensitive.

    ---


    ### fetchValueOptionGroup

    ```
    public static java.lang.String fetchValueOptionGroup(java.lang.String toSelect,
                                                         java.lang.String[] options)
    ```

    :   Returns a selected option group with the toSelect selected if present in the String[].
        This method assumes that an id is at the beginning of some options (ie options[i] =
        "7: Human: modern"). The method will strip off the "7:" and display "Human: modern"
        but set the return value as the original options[i]. This is just a good way to hide the
        return id number from the user.

    ---


    ### fetchMultipleOptionGroup

    ```
    public static java.lang.String fetchMultipleOptionGroup(java.lang.String[] toSelect,
                                                            java.lang.String[] options)
    ```

    :   Returns a selected option group with the toSelect selected if present in the String[]
        of options. Wiggle room allowed in toSelect, checks for blanks too.
        Set toSelect ==null if you just want to list the options.

    ---


    ### fetchCheckGroup

    ```
    public static java.lang.String fetchCheckGroup(java.lang.String name,
                                                   java.lang.String toCheck,
                                                   java.lang.String[] boxes)
    ```

    :   Returns a selected check box group with the toSelect selected if present in the String[]

    ---


    ### fetchRadioGroup

    ```
    public static java.lang.String fetchRadioGroup(java.lang.String name,
                                                   java.lang.String toCheck,
                                                   java.lang.String[] boxes)
    ```

    :   Returns a selected radio group with the toSelect selected if present in the String[]

    ---


    ### fetchRadio

    ```
    public static java.lang.String fetchRadio(java.lang.String name,
                                              java.lang.String value,
                                              java.lang.String potential)
    ```

    :   Returns a radio input line with CHECKED if the value is equal to the potential

    ---


    ### fetchRadioGroupSubmit

    ```
    public static java.lang.String fetchRadioGroupSubmit(java.lang.String name,
                                                         java.lang.String toCheck,
                                                         java.lang.String[] boxes,
                                                         java.lang.String nbsp)
    ```

    :   Returns a selected radio group with the toSelect selected if present in the String[]
        Also throws in a little javascript to submit the form if the button is clicked.
        Add a name = 'form' attribute to the

    ---


    ### populateBean

    ```
    public static void populateBean(java.lang.Object bean,
                                    javax.servlet.http.HttpServletRequest request)
    ```

    :   Fills in all the params of a bean.


    ---


    |  |  |  |
    | --- | --- | --- |
    | **Overview** \| **Package** \| **Class** \| **Use** \| **Tree** \| **Deprecated** \| **Index** \| **Help** | |  |
    | **PREV CLASS**   **NEXT CLASS** | **FRAMES**    **NO FRAMES**     **All Classes** |  |
    | SUMMARY: NESTED \| FIELD \| CONSTR \| METHOD | DETAIL: FIELD \| CONSTR \| METHOD |  |


    ---
